# Optimization of Pt-C Deposits by Cryo-FIBID: Substantial Growth Rate Increase and Quasi-Metallic Behaviour

**DOI:** 10.3390/nano10101906

**Published:** 2020-09-24

**Authors:** Alba Salvador-Porroche, Soraya Sangiao, Patrick Philipp, Pilar Cea, José María De Teresa

**Affiliations:** 1 Instituto de Nanociencia y Materiales de Aragón (INMA), CSIC-Universidad de Zaragoza, 50009 Zaragoza, Spain; asalvador@unizar.es (A.S.-P.); sangiao@unizar.es (S.S.); pilarcea@unizar.es (P.C.); 2 Laboratorio de Microscopías avanzadas (LMA), Universidad de Zaragoza, 50018 Zaragoza, Spain; 3 Departamento de Física de la Materia Condensada, Facultad de Ciencias, Universidad de Zaragoza, 50009 Zaragoza, Spain; 4 Advanced Instrumentation for Ion Nano-Analytics (AINA), MRT Department, Luxembourg Institute of Science and Technology (LIST), 41 rue du Brill, L-4422 Belvaux, Luxembourg; patrick.philipp@list.lu; 5 Departamento de Química Física, Facultad de Ciencias, Universidad de Zaragoza, 50009 Zaragoza, Spain

**Keywords:** nanolithography, focused ion beam induced deposition, cryogenic conditions, Pt-based deposits, metal layers, semiconductor industry

## Abstract

The Focused Ion Beam Induced Deposition (FIBID) under cryogenic conditions (Cryo-FIBID) technique is based on obtaining a condensed layer of precursor molecules by cooling the substrate below the condensation temperature of the gaseous precursor material. This condensed layer is irradiated with ions according to a desired pattern and, subsequently, the substrate is heated above the precursor condensation temperature, revealing the deposits with the shape of the exposed pattern. In this contribution, the fast growth of Pt-C deposits by Cryo-FIBID is demonstrated. Here, we optimize various parameters of the process in order to obtain deposits with the lowest-possible electrical resistivity. Optimized ~30 nm-thick Pt-C deposits are obtained using ion irradiation area dose of 120 μC/cm^2^ at 30 kV. This finding represents a substantial increment in the growth rate when it is compared with deposits of the same thickness fabricated by standard FIBID at room temperature (40 times enhancement). The value of the electrical resistivity in optimized deposits (~4 × 10^4^ µΩ cm) is suitable to perform electrical contacts to certain materials. As a proof of concept of the potential applications of this technology, a 100 µm × 100 µm pattern is carried out in only 43 s of ion exposure (area dose of 23 μC/cm^2^), to be compared with 2.5 h if grown by standard FIBID at room temperature. The ion trajectories and the deposit composition have been simulated using a binary-collision-approximation Monte Carlo code, providing a solid basis for the understanding of the experimental results.

## 1. Introduction

Emerging and new technologies are required in the field of micro/nano-electronics for improved contacts and interconnects, new architectures and faster processing [1]. Focused Ion Beam Induced Deposition (FIBID) under cryogenic conditions (Cryo-FIBID) is an emerging nanolithography technique with great potential in this endeavor, due to its high resolution and reduced processing time [2]. In standard FIBID technique, a precursor gas is delivered by means of a gas injection system (GIS) onto the substrate, which is maintained at room temperature. The ion beam irradiation decomposes the precursor monolayer adsorbed on the substrate, forming a deposit that grows in thickness provided that the flux of precursor gas continues. Given the high resolution of the technique in the sub-100 nm range, and the availability of functional deposits, FIBID is an important nanolithography technique for applications in circuit edit [3], mask repair [4], electrical nanocontacts [5], nano-optics [6], superconductivity [7], magnetism [8], etc. Despite these relevant applications and potential of this technique, standard FIBID implies long processing times which open the door to new improvements of this method in order to decrease the ion dose (i.e., the processing time) and also to prevent side effects caused by a high ion irradiation.

In contrast to the standard FIBID technique, in the Cryo-FIBID method a condensed layer of precursor molecules is obtained by cooling the substrate below the condensation temperature of the gaseous precursor material. This condensed layer is irradiated with ions according to a desired pattern and, subsequently, the substrate is heated above the precursor condensation temperature, revealing the deposits with the shape of the exposed pattern. So far, Cryo-FIBID has only been demonstrated using the W(CO)_6_ precursor [2]. In such previous work, it was shown that the optimum irradiation dose is 50 μC/cm^2^, resulting in metallic W-C deposits with ρ ≈ 800 µΩcm. W-C deposits grown by Cryo-FIBID are particularly useful because of the ultrafast growth of metallic deposits (600 times faster than the standard FIBID of W(CO)_6_ at room temperature), and are applied to grow electrical contacts to nanowires [9] and circuit editing (Sangiao et al., unpublished results).

In the present work, we address ourselves to the investigation of Cryo-FIBID using methylcyclopentadienyl trimethyl platinum as precursor, abbreviated henceforward as (CH_3_)_3_Pt(CpCH_3_). Pt-based precursors like (CH_3_)_3_Pt(CpCH_3_) are widely used in standard FIBID processes at room temperature given the high growth rate and quasi-metallic behavior of the deposits [10]. They are used as protective layer for cross-sectional imaging and lamella preparation [11], electrical contacting [12], plasmonics [13], tips for atomic force microscopy [14] and for scanning electrochemical microscopy [15], etc. The deposits grown by means of the (CH_3_)_3_Pt(CpCH_3_) precursor using standard FIBID processes at room temperature contain typically 20–30% of Pt in atomic % and have electrical resistivity of 10^5–^10^6^ µΩ cm for 30–40 nm thickness [10]. The aim of the present work is two-fold: a) to optimize the growth of Pt deposits by Cryo-FIBID, finding appropriate process parameters to obtain, simultaneously, a fast growth rate and a low electrical resistivity, and b) to provide theoretical support to the obtained results and a better understanding of the growth mechanisms involved. The viability of Pt deposition by Cryo-FIBID is reinforced by previous investigations that have demonstrated that focused electron beam irradiation under cryogenic conditions (Cryo-FEBID) of the (CH_3_)_3_Pt(CpCH_3_) precursor is feasible [16]. Additionally, recent theoretical work has emphasized the advantages of ion versus electron precursor decomposition under cryogenic conditions for the metallic content of the deposits [17].

## 2. Materials and Methods

For the present experiments, a Nova NanoLab 200 Dual Beam instrument (FEI Company, Hillsboro, OR, USA) equipped with a cryo-setup module (PPT2000 from Quorum Technologies, Puslinch, ON, Canada), which enables substrate temperatures down to −155 ± 5 °C, has been used. This equipment combines a vertical 30 kV field-emission electron column with a tilted Ga-based 30 kV ion column. These columns are located at 52° from each other and have a coincident point, 5 mm away from the electron-column pole, where the electron and ion beams intersect [18].

An individual gas injection system (GIS) through which (CH_3_)_3_Pt(CpCH_3_) gas is delivered to the chamber is needed for Pt deposition. The substrate (Si or Si/SiO_2_ for electrical measurements) is cooled from 25 °C down to −100 °C in 10 min but prior to this, nitrogen gas should be purged for at least 15 min [9]. As a result of cooling the substrate to carry out the deposition at cryogenic conditions, a (CH_3_)_3_Pt(CpCH_3_) condensed layer is formed onto the surface. After irradiation, the substrate is heated from −100 °C to 50 °C in steps of 10 °C and remains at this temperature for 15 min. If one wants to estimate the total experimental time, the time used for purging, cooling and heating (around 1 h) has to be added to the irradiation time. Normally, several structures are created in one experimental run in order to save equipment time.

Decomposition of condensed layer by focused-ion-beam irradiation was studied at two different ion beam energies (5 keV and 30 keV) and the dwell time was fixed at 200 ns. For structures with smaller areas (~25 µm^2^), an ion beam current of 1 pA and a pitch of 6 nm were used, whereas Pt deposits with larger areas (~1400 µm^2^) were grown by using an ion beam current and a pitch of 10 pA and 30 nm, respectively.

In order to optimize the thickness of the condensed layer, the GIS valve opening time was varied between ~5 s and ~10 s, the distance between the GIS end and substrate was varied between 10 mm and 12 mm and the precursor reservoir temperature was varied in the range from 35 °C to 44 °C. Pt-based deposits were grown by Cryo-FIBID in 5 × 5 µm^2^ areas on Si substrates that were previously ultrasonicated in acetone for 15 min and in isopropanol for 5 min.

For current-versus-voltage four-probe electrical measurements, a Helios NanoLab 650 Dual Beam instrument (FEI Company, Hillsboro, OR, USA) has been used. The electrical characterization was performed by using four electrical microprobes (Kleindiek Nanotechnik GmbH, Reutlingen, Germany) placed inside the chamber for in-situ measurements. A dc electrical current was injected on the two external contacts using a 6221 DC current source (Keithley Instruments, Cleveland, OH, USA), while the voltage drop across the two inner microprobes was measured with a 2182A nanovoltmeter (Keithley Instruments, Cleveland, OH, USA), both Keithley devices are connected to the electrical microprobes via a chamber feedthrough. For this study, Pt-C Cryo-FIBID deposits with total area of 1400 µm^2^ were grown on Si substrates with a 285 nm-thick SiO_2_ layer on top, previously ultrasonicated in acetone and then in isopropanol.

Comparison analysis by energy-dispersive X-ray spectroscopy (EDS) were carried out using an Inspect F-50 SEM (FEI Company, Hillsboro, OR, USA) equipped with an INCA 350 detector (Oxford Instruments, Abingdon, United Kingdom). Furthermore, for the investigation of the composition along the deposit thickness, Scanning Transmission Electron Microscopy (STEM) imaging and EDS studies were performed by means of an Analytical Titan Low-Base operated at 300 keV (FEI Company, Hillsboro, OR, USA), the energy resolution of the EDS experiments was ~125 eV. For that, lamellae were lifted out onto Cu TEM grids after deposits were grown under optimized conditions and protected with W-FEBID and Pt-FIBID.

Insights on the interaction of the Ga^+^ ions with the precursor molecules have been obtained by BCA-based (binary collision approximation) Monte Carlo (MC) simulations using the software code with the name SDTRIMSP [19] that allows for the modelling of dynamic processes and sample evolution with dose. In the current work, interatomic interactions are described by the KrC potential, the electronic stopping by the Oen-Robinson model, and integration is carried out by the Gauss-Mehler method with 16 pivots. The surface binding energy is calculated by $sbe\left(i,\;j\right)=0.5\left(Es_{i}+Es_{j}\right)$, where sbe is the surface binding energy for the current target, and $Es_{i}$ is the atomic surface binding energy of species i. The surface binding energy of species i is calculated for any combination of Ga, Pt, C, H and Si [19]. For the different species, the atomic densities have been taken identical to the bulk values, so that the density of the precursor layer in the simulations might be above the value in the experiments. As the experimental diffusion coefficients of the different species are not known, this effect has been neglected in the modelling, although SDTRIMSP is capable of taking it into account [20,21]. Especially for hydrogen, this can lead to an overestimation of the concentrations after irradiation. For the displacement energies, the default values from the tables provided with the code have been taken: 12 eV for Ga, 33 eV for Pt, 25 eV for C, 0.5 eV for H, and 13 eV for Si. The precursor layer has a thickness of 30 nm and Ga^+^ irradiation has been simulated for the impact energies 5 keV and 30 keV at normal incidence, up to doses of 1000 µC/cm^2^ and 300 µC/cm^2^, respectively. To determine the average implantation depth on the original samples, some simulations were also carried out in the static regime.

## 3. Results

### 3.1. Formation of a Homogeneous (CH_3_)Pt(CpCH_3_) Condensed Layer with Thickness of 20–40 nm

In this section, the different parameters used upon the deposition process have been optimized to obtain a homogeneous precursor condensed layer with the appropriate thickness for the subsequent Ga^+^ irradiation. The results corresponding to this section have been obtained by growing Pt-C cryo-deposits in 5 × 5 µm^2^ areas on a Si substrate and using an ion area dose equal or greater than 36 µC/cm^2^. First, we used the standard (CH_3_)_3_Pt(CpCH_3_) GIS temperature of 44 °C, GIS-substrate working distance of 10 mm and GIS valve aperture time of 10 s. These parameters were later modified following optimization procedures.

The optimization of the substrate temperature was the first step, aiming to form a homogeneous (CH_3_)_3_Pt(CpCH_3_) condensed layer. For that, the irradiation experiments were conducted under substrate temperature of −50 °C, −75 °C and −100 °C. The Pt-C cryo-deposit grown when the substrate temperature was −100 °C presents the most homogeneous morphology, meanwhile some voids and a higher roughness layer are found when the substrate was kept at −50 °C and −75 °C (Figure S1 in Supplementary File). As a consequence, we decided to use a substrate temperature of −100 °C in the next optimization experiments. The optimization of the remaining parameters (working distance, GIS aperture time and precursor temperature) had as an objective to find the appropriate thickness value of the precursor condensed layer. In Cryo-FIBID, an appropriate thickness value for the precursor condensed layer is one that is similar to the ion penetration length. If the thickness of the condensed layer is higher than the ion penetration length, the condensed layer can be lift-off during the heating process to room temperature. If the thickness of the condensed layer is lower than the ion penetration length, a large part of the ion energy would be delivered in the substrate, entailing the need of a higher ion dose to decompose the precursor. In order to find out the appropriate range of thicknesses for the condensed layer, simulations were carried out to investigate the Ga^+^ penetration length, as described hereafter.

Bombardment of the precursor layer on a Si substrate with Ga^+^ ions was simulated to find a suitable range of thickness of our deposits. Figure S2 in Supplementary File shows that the average penetration depth for a 30 nm thick precursor film is of 21 nm for 5 keV Ga^+^ and 44 nm for 30 keV Ga^+^ ions, meaning that for 5 keV most ions stop in the precursor layer and for 30 keV the majority of ions becomes implanted in the Si substrate. Simulations of various individual trajectories under 5 keV and 30 keV Ga^+^ bombardment are shown in Figure S3 in the Supplementary File, with different scenarios of the types of collision cascades obtained. Given the results of the simulations, we aimed to control the condensed layer thickness in the 20 nm to 40 nm range.

**Figure 1:**
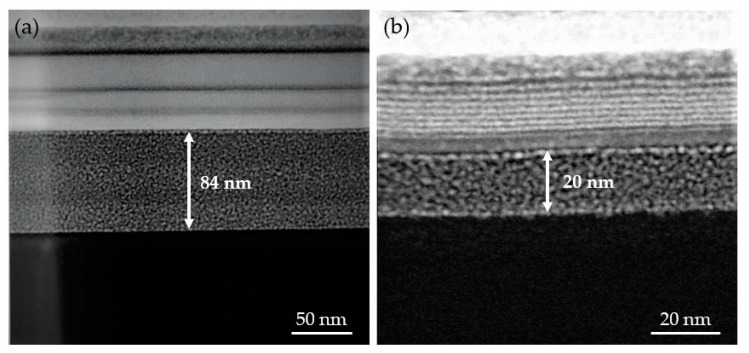
Scanning Transmission Electron Microscopy (STEM) images corresponding to two different Pt-C cryo-deposits grown at substrate temperature of −100 °C and 30 keV ion beam voltage under: (**a**) Non optimized and (**b**) optimized conditions with respect to the GIS opening time, the GIS-substrate distance and the GIS precursor temperature (see the main text for the exact deposition conditions in both cases). The Ga^+^ irradiation area doses used for the 84 nm-thick and 20 nm-thick deposits were 144 μC/cm^2^ and 120 μC/cm^2^, respectively.

The first experiments with GIS temperature of 44 °C, working distance of 10 mm, GIS valve aperture time of 10 s and substrate temperature of −100 °C indicated that the cryo-deposits had a thickness higher than the targeted one. In order to approach the optimal thickness range (20 nm to 40 nm), the GIS valve opening time was decreased to 5 s, which still produced a thick condensed layer of 84 nm, as shown in Figure 1a. In order to achieve the 20 nm–40 nm thickness range, the total flux of gas precursor reaching the substrate needed to be reduced. With that aim, the GIS-substrate working distance was increased to 12 mm (the highest possible value in our equipment) and the GIS temperature was reduced to deliver less precursor [22]. When the GIS temperature was reduced from 44 °C down to 35 °C, the targeted condensate thickness (20–40 nm) was finally attained, as shown in Figure 1b.

### 3.2. Electrical Characterization of Pt-C Cryo-FIBID Deposits

**Figure 2:**
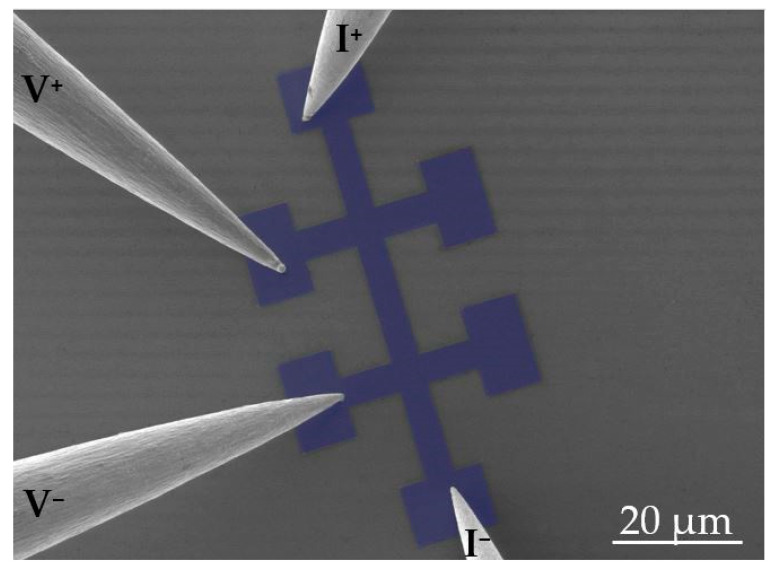
Artificially colored Scanning Electron Microscopy (SEM) micrograph of one of the devices electrically measured at room temperature with the four probes. These Pt-C Cryo-FIBID deposits had an area of ~1400 µm^2^ and the irradiation time was ~5 min, which corresponds to an ion dose of 210 µC/cm^2^ (an ion beam current of 10 pA was used).

The electrical characterization of the Pt-C Cryo-FIBID deposits and the optimization of the Ga^+^ area dose in relation to the resistivity were carried out by using four electrical microprobes at room temperature. The investigated deposits were grown on Si/SiO_2_ substrates under the optimized conditions (substrate temperature: –100 °C, aperture of injector valve: 5 s, working distance: 12 mm and GIS temperature: 35 °C) and according to the sample geometry shown in Figure 2.

The ion beam parameters for the fabrication of these Pt-C cryo-deposits were 10 pA of ion beam current, 30 nm of pitch, 200 ns of dwell time and 0 % of X and Y overlap. These deposits were carried out at two different Ga^+^ beam energies (5 keV and 30 keV) in order to investigate the influence that this energy has on the ion area dose required for their optimized behavior. Pt-based deposits were grown with ion area doses ranging from 160 µC/cm^2^ up to 930 µC/cm^2^ for 5 keV Ga^+^ and from 100 µC/cm^2^ up to 230 µC/cm^2^ for 30 keV Ga^+^.

**Figure 3:**
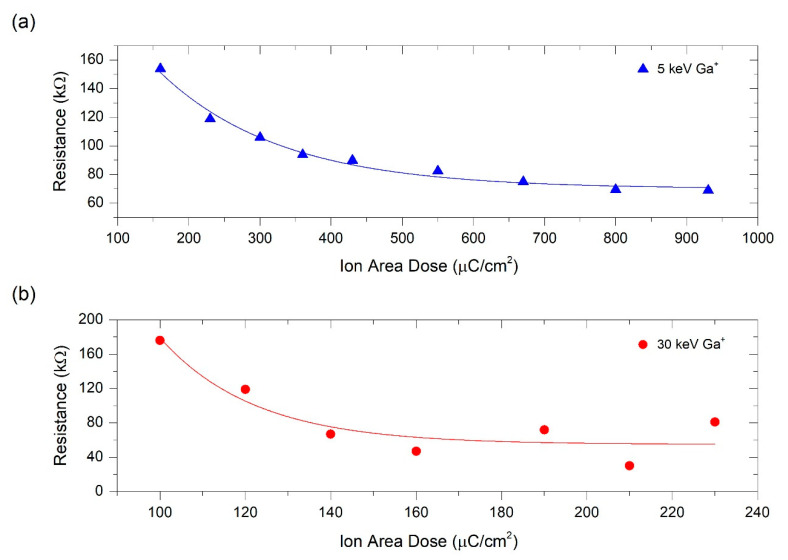
Electrical resistance of Pt-C deposits as a function of the ion area dose used in the Cryo-FIBID process. (**a**) Resistance values corresponding to deposits grown at 5 keV Ga^+^; (**b**) Resistance values corresponding to deposits grown at 30 keV Ga^+^.

Electrical characterization consisted of performing a current sweep, applying the current through the two outer microprobes, while measuring the voltage drop across the two inner microprobes (see Figure 2). A linear behavior was observed in the current-vs-voltage characteristics measured on these samples (see Figures S4 and S5 in Supplementary File). Considering the Ohm’s law, from linear fits to the *I-V* data, electrical resistance can be calculated and represented as a function of the Ga^+^ ion area dose (see Figure 3).

The resistance values achieved for both ion beam energies indicate that the electrical resistance decreases as a function of the Ga^+^ ion area dose, tending to saturation beyond a certain value. The electrical resistivity was calculated after determining the transversal area and length of Pt-C deposits. Width and length were directly measured directly in the Helios NanoLab 650 Dual Beam instrument with the SEM images of each deposit, whereas the thickness was determined using a KLA Tencor P-6 (KLA-Tencor, Milpitas, CA, USA) profilometer (see Tables S1 and S2 in Supplementary File). The thickness varied randomly from 20 nm to 41 nm, which is explained by the slightly changing growth conditions: the process chamber pressure and existing residual species, the total precursor flux delivered during the manual opening and closing of the valve, the distance (in x-y-z) between the GIS end and the substrate, the substrate surface roughness and cleanliness of the irradiation area and the specific ion irradiation dose. Although we do not know at present which of these factors is the most important one, we suspect that the total precursor flux delivered during the manual opening and closing of the valve is a relevant factor. The error bar in the experimental electrical resistivity was calculated taking into account the instrumental error of the profilometer, which was of 3 nm.

**Figure 4:**
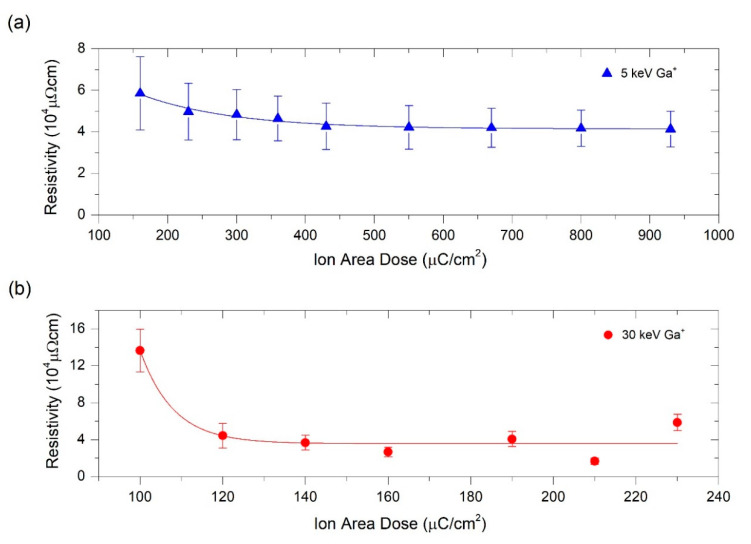
Electrical resistivity of Pt-C deposits as a function of the ion area dose used for their fabrication. (**a**) Resistivity values corresponding to deposits grown at 5 keV Ga^+^; (**b**) Resistivity values corresponding to deposits grown at 30 keV Ga^+^. The main source of the error bar is the thickness indeterminacy owing to the ±3 nm precision of the profilometer.

As shown in Figure 4, the electrical resistivity follows the same trend as the electrical resistance given that the width and length of each deposit remains constant and the thickness varies little around the targeted value of 30 nm. The deposits grown at 5 keV showed a thickness of 25 ± 3 nm and those grown at 30 keV exhibited a thickness of 30 ± 7 nm. As expected, the electrical resistivity saturates at different ion area doses for deposits grown at 5 keV and 30 keV, with electrical resistivity saturations at ~430 µC/cm^2^ and ~120 µC/cm^2^, respectively. In both cases, the lowest electrical resistivity reached is ~4 × 10^4^ µΩ cm.

### 3.3. Compositional Analysis of Pt-C Cryo-FIBID Deposits by EDS

**Figure 5:**
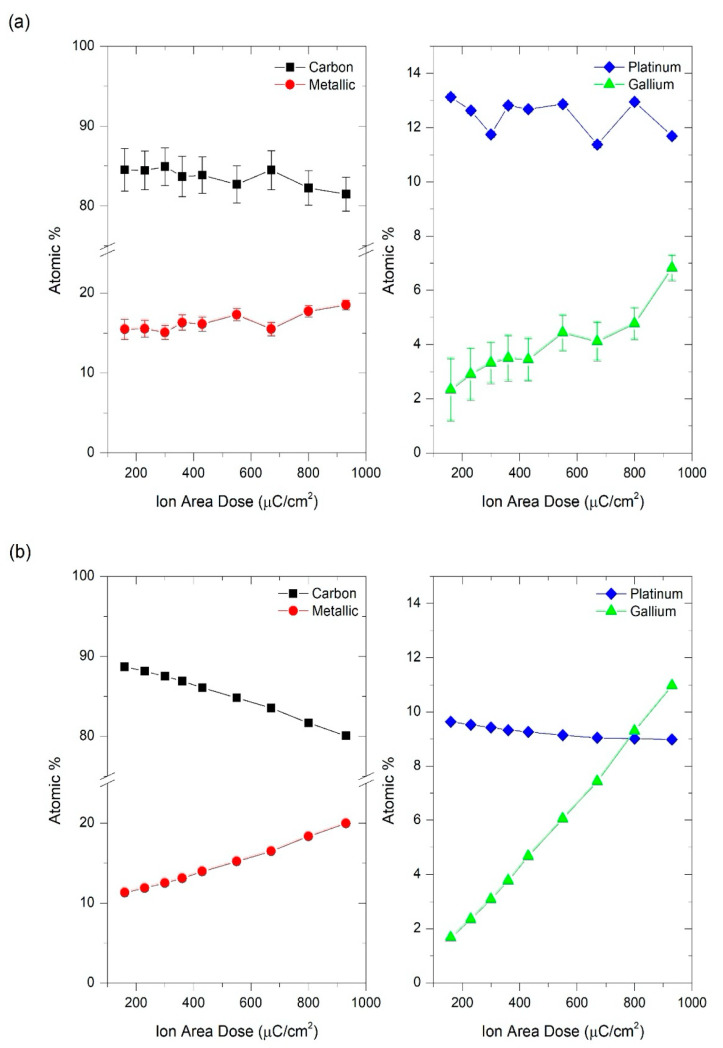
Atomic percentages as a function of the ion area dose of Pt-C cryo-deposits grown at 5 kV. (**a**) Experimental EDS compositional results. Left: carbon and metallic (platinum plus gallium) content. Right: platinum and gallium content; (**b**) Compositional results extracted from MC simulations at a depth of 15.25 nm (center of the deposit). Left: carbon and metallic (platinum plus gallium) content. Right: platinum and gallium content.

**Figure 6:**
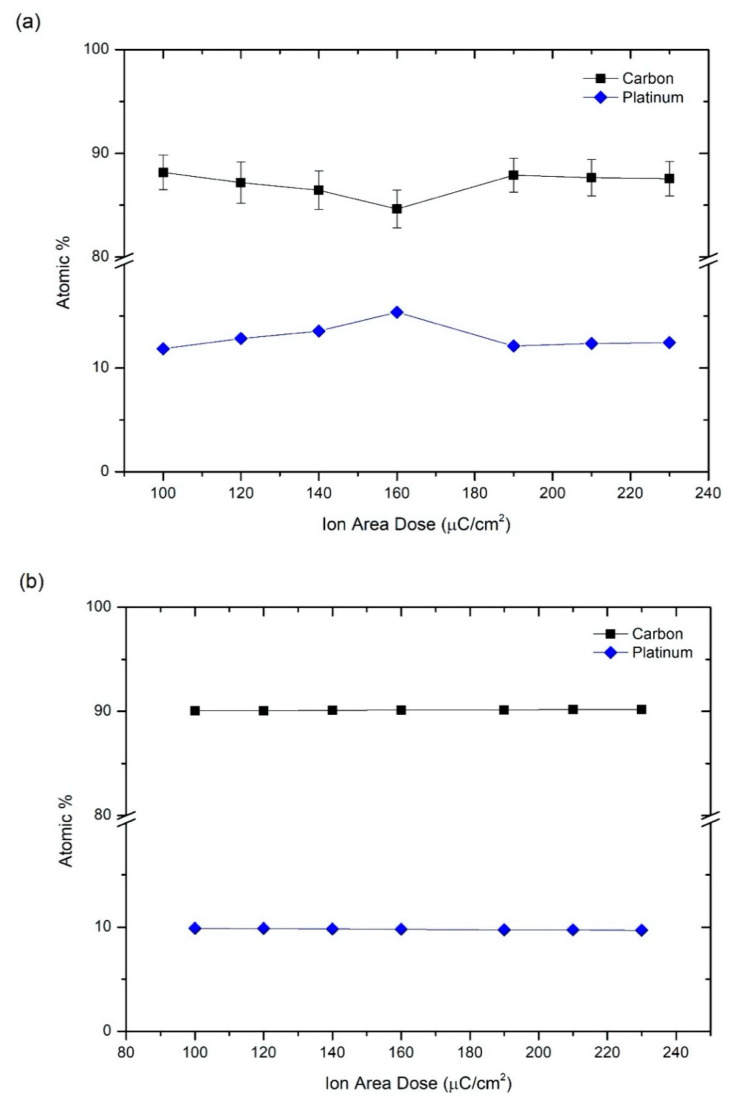
Atomic percentages as a function of the ion area dose of Pt-C cryo-deposits grown at 30 keV Ga^+^. (**a**) Experimental compositional results of deposits grown by Cryo-FIBID technique; (**b**) Compositional results extracted from Monte Carlo simulations at a depth of 15.25 nm.

In order to correlate the resistivity of the deposits as a function of the ion irradiation dose with their metallic content, energy dispersive X-ray spectroscopy (EDS) experiments were performed in selected areas of 12 µm × 6 µm approximately and using an electron voltage of 5 kV. Given that some of the electrons reach the underlying Si/SiO_2_ substrate at this voltage, from the raw data, the background Si and O signals were subtracted. As expected, the remaining peaks in the spectrum reveal the presence of carbon (C), platinum (Pt) and gallium (Ga). The composition in atomic % of cryo-deposits is represented as a function of ion area dose in Figure 5a and Figure 6a for 5 keV and 30 keV Ga^+^ irradiation, respectively. In all cases, an important concentration of carbon (80–90%) is observed due the high content of this element in the platinum-based precursor, (CH_3_)_3_Pt(CpCH_3_). A lower but significant platinum content (10–15%) is also measured. For that reason, we refer to these deposits as Pt-C Cryo-FIBID deposits. In addition, the deposits irradiated under 5 keV show the presence of gallium (2–7%), which is not the case when the deposits are irradiated under 30 keV. In the latter deposits, the Ga content can nevertheless be below the detection limit of the technique.

The experimental compositional results can be compared to data extracted from BCA-based MC simulations at different doses for 5 keV and 30 keV Ga^+^ irradiation, which are shown in Figure 5b and Figure 6b. In the simulations, the largest amount of H remains inside the precursor film. However, its role in the electrical properties is unlikely to be relevant and is not considered in the discussion. In addition, diffusion processes are not taken into account in the MC simulations, so that the real H concentration is certainly lower. Furthermore, the formation of volatile CH_x_ or C_x_H_y_ fragments cannot be modelled but are likely to be emitted in our experiments, leading to experimental Pt/C ratios slightly higher than those predicted by the simulations. At the same time, the simulations predict that the 5 keV Ga^+^ beam should lead to stronger modification of the topmost region of the precursor layer, while the 30 keV Ga^+^ beam should produce a more homogeneous modification of the layer (Figure S6 in the Supplementary File). For both energies, hydrogen atoms account for the largest part of the recoils (Figure S7 in the Supplementary File).

The EDS experiments of deposits grown at 5 keV Ga^+^ (represented in Figure 5a) indicate that gallium is implanted in the deposits in the whole range of ion doses, from 160 µC/cm^2^ to 930 µC/cm^2^, increasing this percentage as the dose is higher. This finding agrees well with the simulations shown in Figure 5b. As a consequence, the total metallic content (platinum plus gallium) increases as a function of the Ga^+^ irradiation dose, in good agreement with the resistivity results shown in Figure 4. Considering the compositional analysis, the electrical resistivity measurements and the processing time, 230 µC/cm^2^ can be considered an optimized ion area dose for the deposits grown at 5 keV.

The EDS experiments of deposits grown at 30 keV Ga^+^ (represented in Figure 6a) show no presence of gallium, which can be explained by the fact that this element is implanted in the substrate and not in the precursor layer, as supported by the simulations. In fact, the simulations predict an atomic concentration of gallium from 0.05 % for the lowest dose (100 μC/cm^2^) up to 0.13% for the highest studied dose (230 μC/cm^2^). Due to these negligible values, only carbon and platinum content are shown in Figure 6b. The optimized Ga^+^ area dose for deposits grown at 30 keV was determined to be 120 µC/cm^2^ as it corresponds to one of the lowest resistivities obtained (4 × 10^4^ µΩ cm).

Once the compositional analysis by EDS of each deposit had been determined, the composition study of the deposits with optimal doses for both 5 keV and 30 keV Ga^+^ irradiation was investigated along the layer thickness. For that, lamellae were extracted from the deposit grown with a 5 keV Ga^+^ 230 μC/cm^2^ irradiation dose and from the deposit fabricated with a 30 keV Ga^+^ 120 μC/cm^2^ irradiation dose, and studied by STEM. As shown in Figures S8 and S9, the deposits grown under optimized conditions at both voltages do not exhibit voids or defects, in addition to having homogenous thickness and low roughness. Also, atomic percentages of Pt, C and Ga in six selected areas of our cryo-deposits are shown in Tables S3 and S4.

## 4. Discussion and Outlook

It was previously found by Bresin et al. that the growth of Pt-cryo-FEBID deposits was optimized with an area dose in the range of 10^3^ μC/cm^2^ [22], resulting in an increment of four orders of magnitude in the growth rate compared to Pt-FEBID deposits grown at room temperature. On the other hand, Pt-FIBID deposits grown at room temperature requires an area dose around 5 × 10^3^ μC/cm^2^ [18] whereas the Pt-Cryo-FIBID deposits investigated here need an area dose in the range of 120 µC/cm^2^ in order to obtain optimized deposits from the point of view of their electrical properties. It means that the growth rate of optimized Pt-based deposits under cryogenic conditions is about 40 times faster than those grown at room temperature. Figure S10 in Supplementary File offers a comparison of different lithography techniques in relation to the required irradiation dose. It is noticed that Cryo-FIBID requires the lowest irradiation dose per area amongst the single-step lithography techniques.

As a result of the increment in the growth rate, the Pt-C cryo-deposits are very promising for different applications in nanotechnology. One application of Pt-C cryo-deposits could be found in the growth of electrical contacts at the micro/nano-scale, as already demonstrated for W-C cryo-deposits [9]. The range of electrical resistivity achievable for the Pt-C cryo-deposits, ~4 × 10^4^ µΩ cm, is suitable to contact semiconductor micro/nano-structures [12], with the advantage of a reduced ion damage due to the low ion dose required. Moreover, Pt-C deposits grown at room temperature have recently been singled out as potential cryogenic resistive thermometers [23], application that could be also explored for Pt-C cryo-deposits. Another potential application of a Pt-C cryo-deposit could be found as a structural material, similarly to the Pt-C deposits grown at room temperature that are used as protective layers for cross-sectional imaging and lamella preparation [11]. In those applications, the enhanced growth rate and the reduced ion-induced damage of Pt-C cryo-deposits are great advantages.

**Figure 7:**
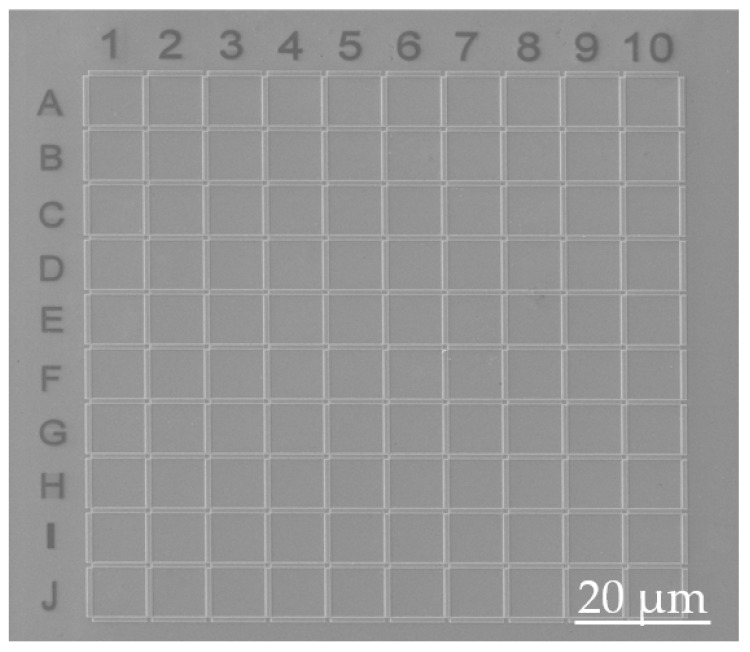
SEM micrograph of a Pt-C cryo-deposit grid-like array formed by 220 rectangles with ~9 µm × 0.9 µm area. The total Ga^+^ irradiation time was only 43 s, which corresponds to an ion area dose of 23 µC/cm^2^. The same array would fabricate by standard FIBID process in 2.5 h. This type of arrays is useful as a reference framework when working with 2D materials, nanowires dispersed from a solution, etc., and to form barriers for cell proliferation in biological studies.

In addition to the applications described in the previous paragraph, in the present work, we present another application of Pt-C cryo-deposits to generate micro/nano-patterned grids. As a proof of concept, a grid-like array of area 100 µm × 100 µm has been grown by using Pt-C Cryo-FIBID deposits, as shown in Figure 7. Such types of arrays are useful to identify the exact position of micro/nano-objects (manually exfoliated materials, dispersed nanowires, etc.) in a substrate or to insert barriers for cell proliferation in biological studies [24] and are commonly created by optical or electron beam lithography, which leave resist residues on the substrate. A total of 220 rectangles with 9 µm × 0.9 µm areas were grown, with a total irradiation time of 43 s, which corresponds to a Ga^+^ ion dose of 23 µC/cm^2^. We have chosen a very low irradiation dose given that in this application an optimized electrical resistivity is not required. The total irradiation time of the arrays was only 43 s, to be compared with 2.5 h if the process is performed at room temperature. In addition, numbers and letters were milled at room temperature on the top and left parts in order to provide references for the sample locations in case that this is needed. Other nanolithography techniques like electron beam lithography (EBL) with polymethyl methacrylate (PMMA) resist is also useful for this kind of applications [25]. The main disadvantages of EBL are the requirement for a higher irradiation dose and the complexity of the technique owing to the need of various lithography steps [25], which in turn may contaminate the substrate with resist residues. Thus, Pt-C cryo-deposits hold great potential for the fabrication of certain micro/nano-structures due to the single-step nature, the fast growth rate and the lack of residues on the substrate.

## 5. Conclusions

In the present work, the growth of Pt-C deposits by Cryo-FIBID is introduced for the first time. The main advantages of Cryo-FIBID are the fast growth rate and the low ion irradiation dose needed in comparison with FIBID at room temperature, as well as the absence of resists. We have shown that the optimization of different parameters (substrate temperature, GIS aperture time, GIS-substrate distance and GIS temperature) leads to suitable deposits in terms of homogeneity and Pt composition, in the range of 10 to 15%. From the electrical characterization, it has been concluded that the lowest resistivity (~4 × 10^4^ µΩ cm) is obtained for deposits grown at 30 keV Ga^+^ with an ion dose of 120 μC/cm² and at 5 keV Ga^+^ with an ion dose of 430 μC/cm². Simulations of the ion trajectories and precursor dissociation support the experimental findings. A grid-like array of rectangles with total area of 100 µm × 100 µm has been grown in only 43 s of ion irradiation, highlighting the great potential of Pt-C Cryo-FIBID as a resist-free fast patterning technique.

## Supplementary Materials

The following are available online at https://www.mdpi.com/2079-4991/10/10/1906/s1; Figure S1: SEM micrographs of the (CH_3_)_3_Pt(CpCH_3_) condensed layers when the substrate temperature was (a) −50 °C, (b) −75 °C, and (c) −100 °C. The scale bar is the same for (a), (b) and (c); Figure S2: Trajectories of implanted Ga^+^ ions (left axis, a different shade of green per trajectory) and distribution of implantation depths (right axis) for (a) 5 keV, and (b) 30 keV. The results have been obtained by SDTRIMSP simulations; Figure S3: Three different scenarios of collision cascades obtained by SDTRIMSP for (a) 5 keV, and (b) 30 keV Ga^+^ irradiation of the precursor layer on top of a Si substrate. Colors of the species: Ga in grey, Si in green, Pt in blue, C in magenta, H in red. Figure S4: Voltage-versus-current (*V-I*) experiments measurements of Pt-C deposits grown at 5 keV Ga^+^ irradiation and under ion area doses from 160 µC/cm^2^ up to 930 µC/cm^2^; Figure S5: Voltage-versus-current (*V-I*) experiments measurements of Pt-C deposits grown at 30 keV Ga^+^ irradiation and under ion area doses from 100 µC/cm^2^ up to 230 µC/cm^2^; Table S1: Data corresponding to dimensions as well as the electrical resistance and resistivity of deposits grown at 5 keV Ga^+^ irradiation; Table S2: Data corresponding to dimensions, electrical resistance and resistivity of deposits grown at 30 keV Ga^+^ irradiation; Figure S6: Composition of the precursor layers versus depth obtained by SDTRIMSP simulations for (a) 5 keV Ga^+^ irradiation at a dose of 930 µC/cm^2^, and (b) 30 keV Ga^+^ irradiation at a dose of 230 µC/cm^2^. The composition of H and C is overestimated as the emission of volatile species, e.g., $H_{2}$, ${\mathrm{CH}}_{y}$, and $C_{x}H_{y}$, is not taken into account in the simulations. For 5 keV, the topmost region of the precursor should be more modified by the Ga^+^ irradiation than for 30 keV Ga^+^ ions; Figure S7: Trajectories of Ga and recoil atoms obtained by SDTRIMSP for 5 keV Ga^+^ (a) projection on *xy* plane, (b) projection on *xz* plane, and 30 keV Ga^+^ (c) projection on *xy* plane, and (d) projection on *xz* plane; Figure S8: STEM images of the Pt-C cryo-deposit grown under optimized conditions at 5 keV Ga^+^ irradiation. The white rectangles correspond to the areas where EDS experiments were carried out to study the composition along the thickness; Table S3: Data corresponding to atomic percentages of C, Pt and Ga of the Pt-C cryo-deposit grown under optimized conditions at 5 keV Ga^+^ irradiation; Figure S9 STEM images of the Pt-C cryo-deposit grown under optimized conditions at 30 keV Ga^+^ irradiation. The white rectangles correspond to the areas where EDS experiments were carried out to study the composition along the thickness; Table S4 Data corresponding to atomic percentages of C, Pt and Ga of the Pt-C cryo-deposit grown under optimized conditions at 30 keV Ga^+^ irradiation; Figure S10: Comparison of charge-particle-based lithography techniques in terms of the required irradiation dose. The Cryo-FIBID deposits are those that require the lower irradiation doses per area among the single-step techniques. Adapted and modified from De Teresa, J.M. et al., Micromachines 2019 [2].
