# Supplementary material for: Optimization of Pt-C Deposits by Cryo-FIBID: Substantial Growth Rate Increase and Quasi-Metallic Behaviour
Source: Nanomaterials (Basel). 2020 Sep 24;10(10):1906. doi: 10.3390/nano10101906 (PMC7600287; doi:10.3390/nano10101906)
Supplement: Supplementary file 1 [file nanomaterials-10-01906-s001.pdf]

Supplementary Information

Optimization of Pt-C deposits by Cryo-FIBID: substantial growth rate increase and quasi-metallic behaviour

Alba Salvador-Porroche [1,2], Soraya Sangiao [1,2,3], Patrick Philipp [4], Pilar Cea [1,2,5] and José María De Teresa [1,2,3,*]

[1] Instituto de Nanociencia y Materiales de Aragón (INMA), CSIC-Universidad de Zaragoza, 50009 Zaragoza, Spain; asalvador@unizar.es
[2] Laboratorio de Microscopías avanzadas (LMA), Universidad de Zaragoza, 50018 Zaragoza, Spain; deteresa@unizar.es
[3] Departamento de Física de la Materia Condensada, Facultad de Ciencias, Universidad de Zaragoza, 50009 Zaragoza, Spain; sangiao@unizar.es
[4] Advanced Instrumentation for Ion Nano-Analytics (AINA), MRT Department, Luxembourg Institute of Science and Technology (LIST), 41 rue du Brill, L-4422 Belvaux, Luxembourg; patrick.philipp@list.lu
[5] Departamento de Química Física, Facultad de Ciencias, Universidad de Zaragoza, 50009 Zaragoza, Spain; pilarcea@unizar.es

* Correspondence: deteresa@unizar.es

Received: date; Accepted: date; Published: date

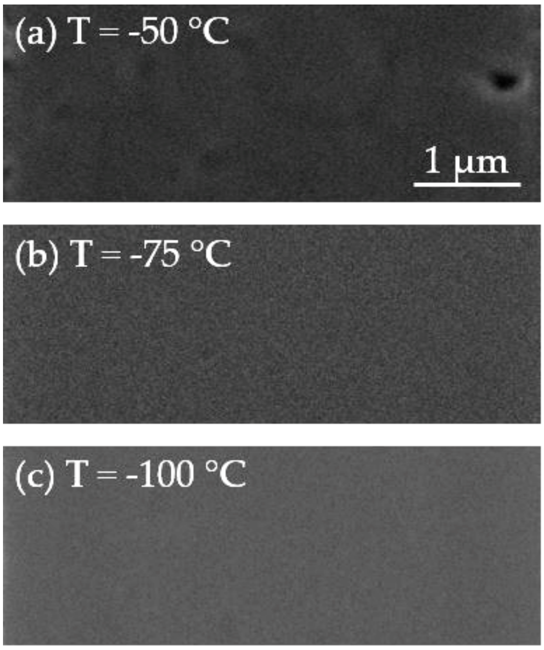

Figure S1. SEM micrographs of the $(CH_3)_3Pt(CpCH_3)$ condensed layers when the substrate temperature was (a) −50 ºC, (b) −75 ºC, and (c) −100 ºC. The scale bar is the same for (a), (b) and (c).

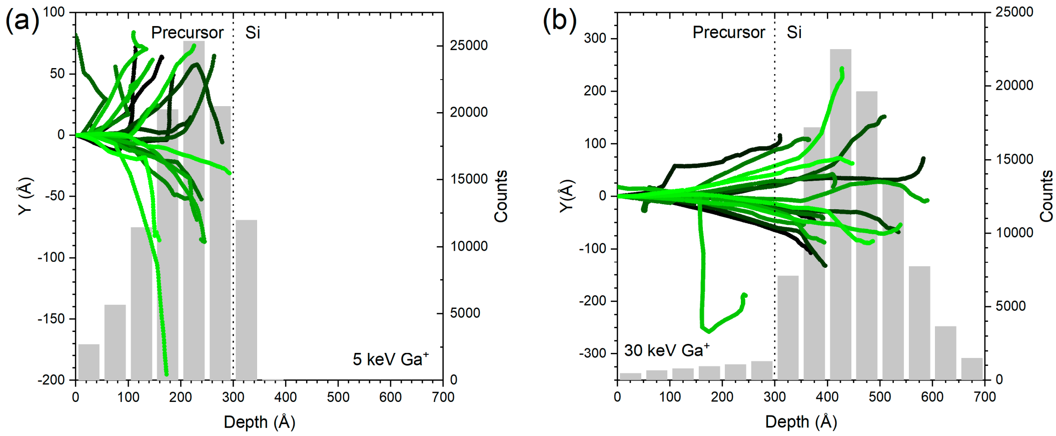

Figure S2. Trajectories of implanted Ga^+ ions (left axis, a different shade of green per trajectory) and distribution of implantation depths (right axis) for (a) 5 keV, and (b) 30 keV. The results have been obtained by SDTRIMSP simulations.

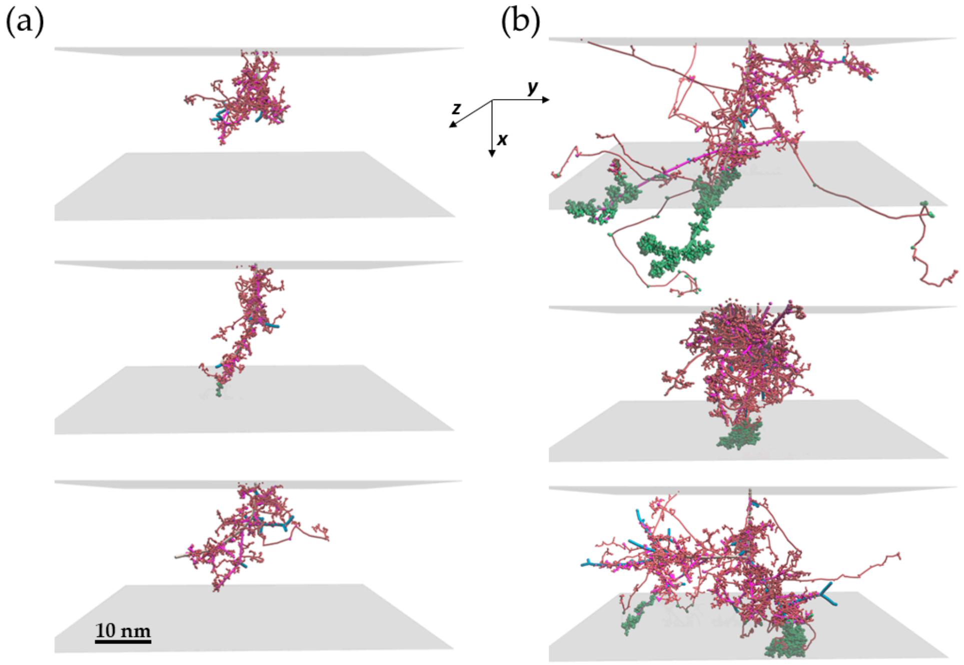

Figure S3. Three different scenarios of collision cascades obtained by SDTRIMSP for (a) 5 keV, and (b) 30 keV Ga^+ irradiation of a 30 nm-thick precursor layer on top of a Si substrate. Colors of the species: Ga in grey, Si in green, Pt in blue, C in magenta, H in red.

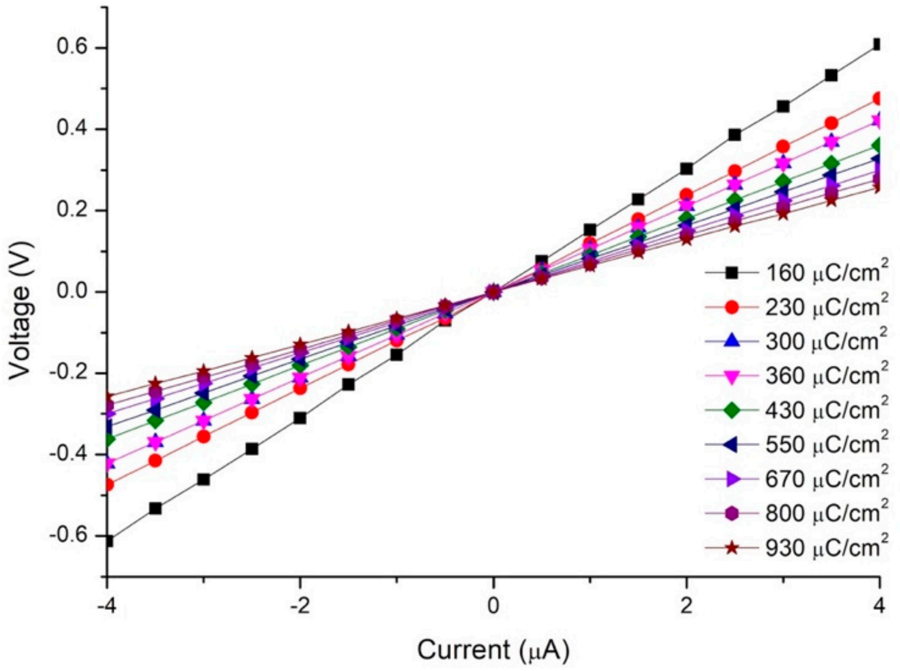

Figure S4. Voltage-versus-current (*V*-*I*) measurements of Pt-C deposits grown at 5 keV Ga^+ irradiation and under ion area doses from 160 μC/cm² up to 930 μC/cm².

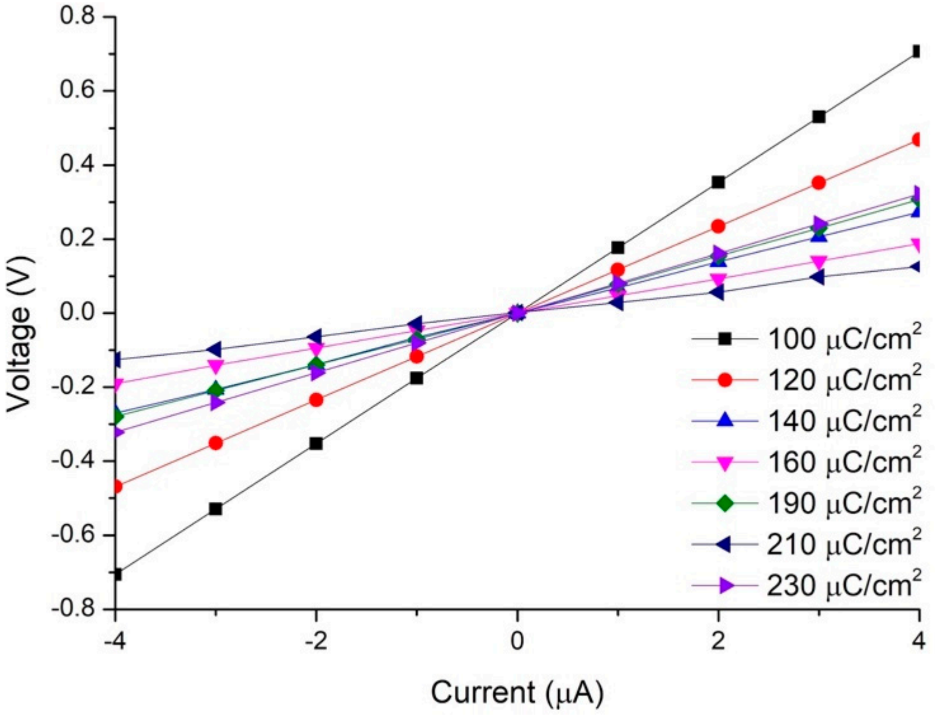

Figure S5. Voltage-versus-current (*V*-*I*) measurements of Pt-C deposits grown at 30 keV Ga^+ irradiation and under ion area doses from 100 μC/cm² up to 230 μC/cm².

Table S1. Data corresponding to dimensions as well as the electrical resistance and resistivity of deposits grown at 5 keV Ga^+ irradiation.

**Ion Dose ($\mu C/cm^2$)**	**Width (μm)**	**Thickness (10^{-7} cm)**	**Length (μm)**	**Resistance (10^9 $\mu\Omega$)**	**Resistivity[1] (10^4 $\mu\Omega cm$)**
160	5.01	20	26.3	154	5.86 ± 1.8
230	5.01	22	26.3	119	4.98 ± 1.4
300	5.01	24	26.3	106	4.84 ± 1.2
360	5.01	26	26.3	94	4.65 ± 1.1
430	5.44	23	26.3	90	4.27 ± 1.1
550	5.44	25	26.5	82.5	4.22 ± 1.0
670	5.44	27	26.2	75	4.20 ± 0.93
800	5.44	29	26.2	69.5	4.18 ± 0.86
930	5.76	29	26.2	65	4.14 ± 0.85

[1]Electrical resistivity was calculated considering the instrumental error of profilometer of 3 nm approximately.

Table S2. Data corresponding to dimensions, electrical resistance and resistivity of deposits grown at 30 keV Ga^+ irradiation.

**Ion Dose ($\mu C/cm^2$)**	**Width (μm)**	**Thickness (10^{-7} cm)**	**Length (μm)**	**Resistance (10^9 $\mu\Omega$)**	**Resistivity (10^4 $\mu\Omega cm$)**
100	4.07	41	21.5	176	13.7 ± 2.3
120	4.07	20	21.4	119	4.44 ± 1.3
140	4.21	28	21.4	67	3.68 ± 0.79
160	4.14	30	21.9	47	2.67 ± 0.53
190	4.07	30	21.5	72	4.09 ± 0.82
210	4.00	30	21.4	30	1.68 ± 0.34
230	4.43	40	24.4	81	5.87 ± 0.88

[1]Electrical resistivity was calculated considering the instrumental error of profilometer of 3 nm approximately.

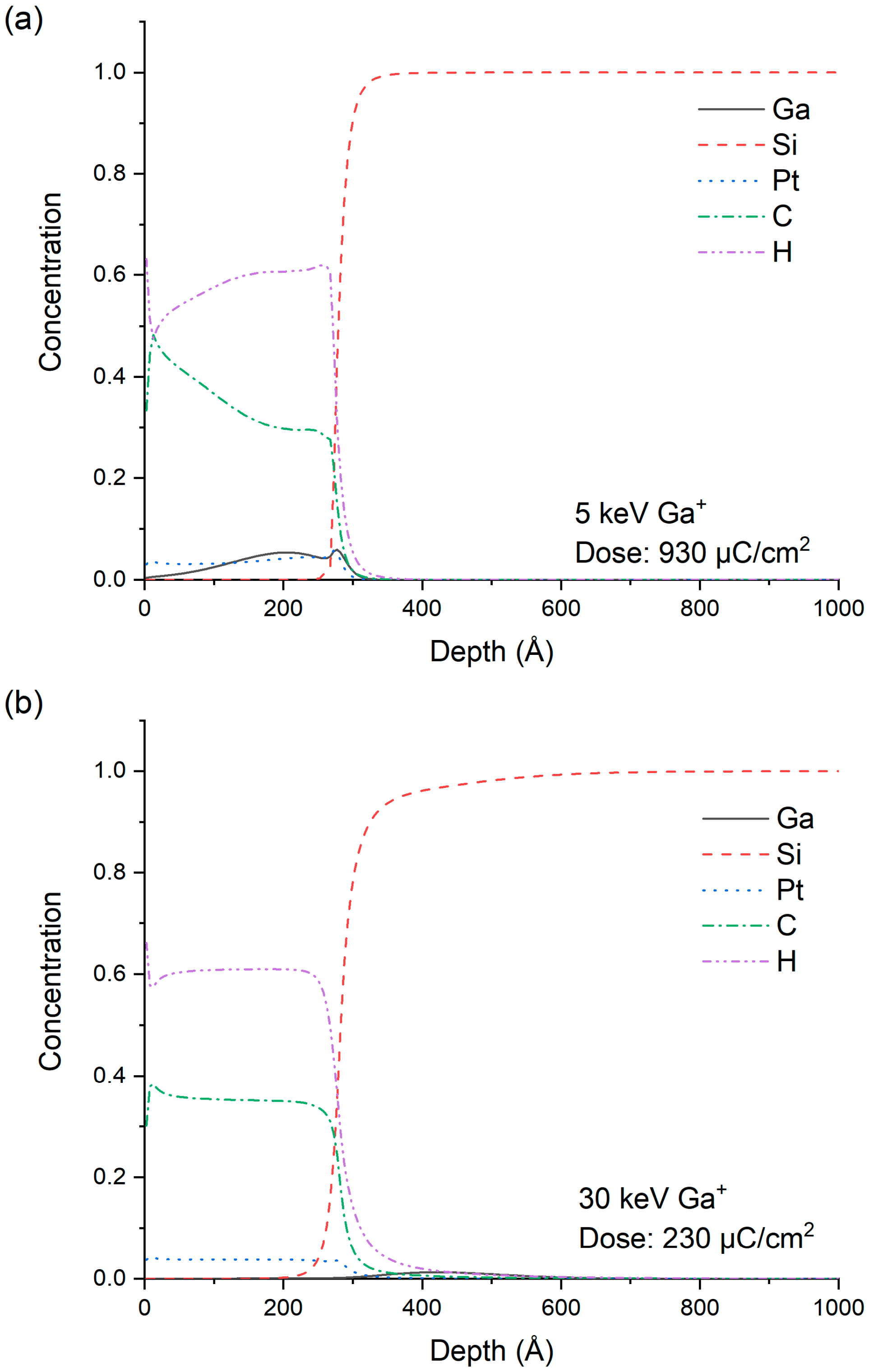

Figure S6. Composition of the precursor layers versus depth obtained by SDTRIMSP simulations for (a) 5 keV Ga^+ irradiation at a dose of 930 μC/cm^2, and (b) 30 keV Ga^+ irradiation at a dose of 230 μC/cm^2. The composition of H and C is overestimated as the emission of volatile species (e.g. H_2, CH_y, and C_xH_y) is not taken into account in the simulations. For 5 keV, the topmost region of the precursor should be more modified by the Ga^+ irradiation than for 30 keV Ga^+ ions.

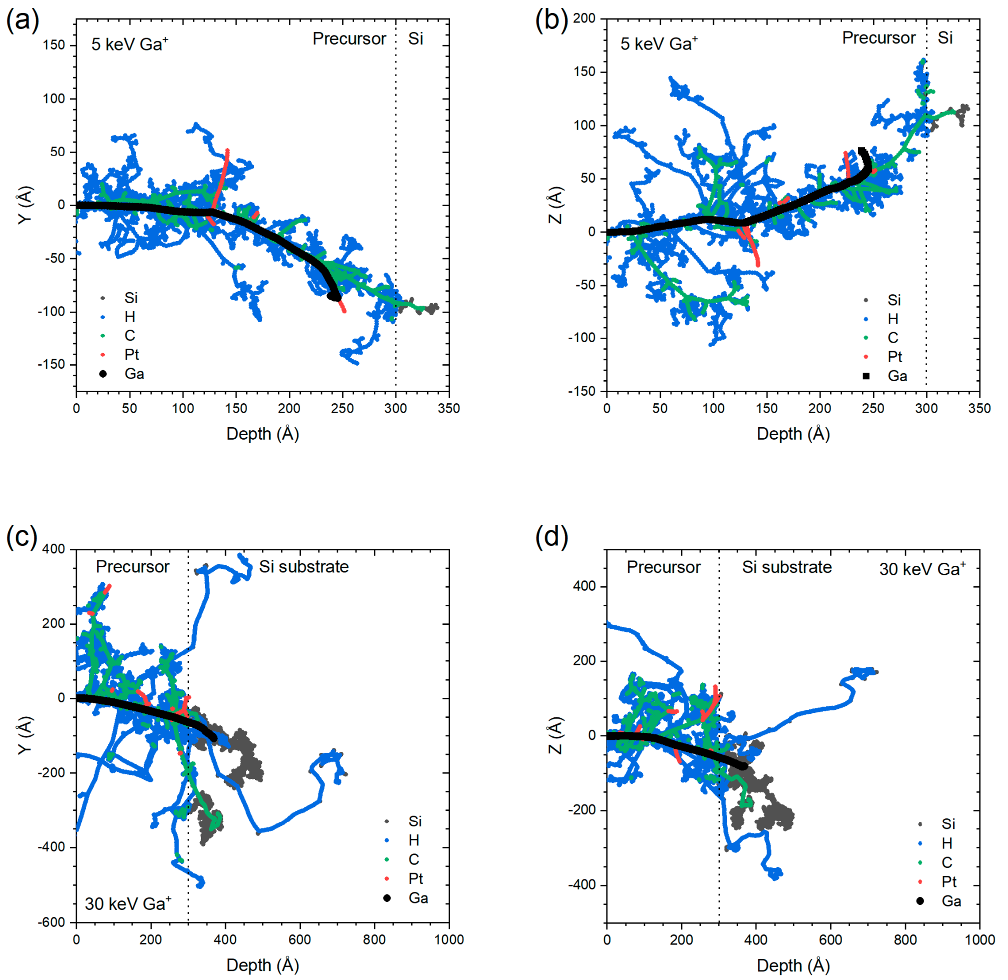

Figure S7. Trajectories of Ga and recoil atoms obtained by SDTRIMSP for 5 keV Ga^+ (a) projection on *xy* plane, (b) projection on *xz* plane, and 30 keV Ga^+ (c) projection on *xy* plane, and (d) projection on *xz* plane.

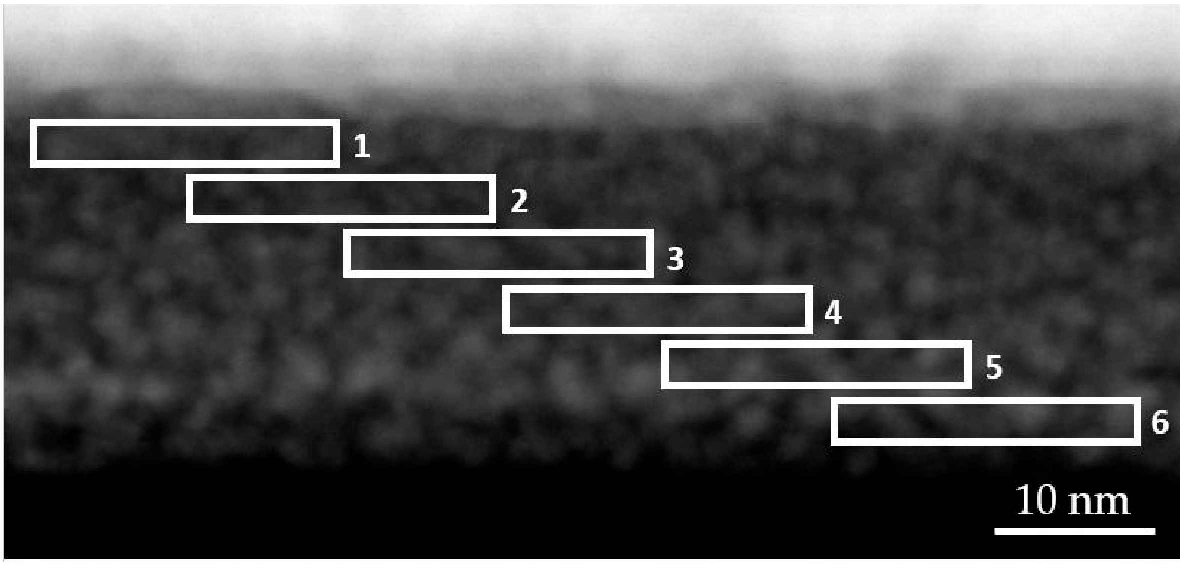

Figure S8. STEM images of the Pt-C cryo-deposit grown under optimized conditions at 5 keV Ga^+ irradiation. The white rectangles correspond to the areas where EDS experiments were carried out to study the composition along the thickness.

Table S3. Data corresponding to atomic percentages of C, Pt and Ga of the Pt-C cryo-deposit grown under optimized conditions at 5 keV Ga^+ irradiation.

Area	C (atomic %)	Pt (atomic %)	Ga (atomic %)
1	77.42 ± 1.32	11.77 ± 1.60	7.79 ± 0.81
2	81.04 ± 1.29	12.36 ± 1.49	6.59 ± 0.70
3	77 ± 1.40	17.65 ± 1.91	5.34 ± 0.72
4	67.32 ± 1.26	25.70 ± 2.25	6.96 ± 0.82
5	70.53 ± 1.28	25.70 ± 2.14	3.76 ± 0.61
6	83.54 ± 1.69	12.80 ± 1.95	3.64 ± 0.76

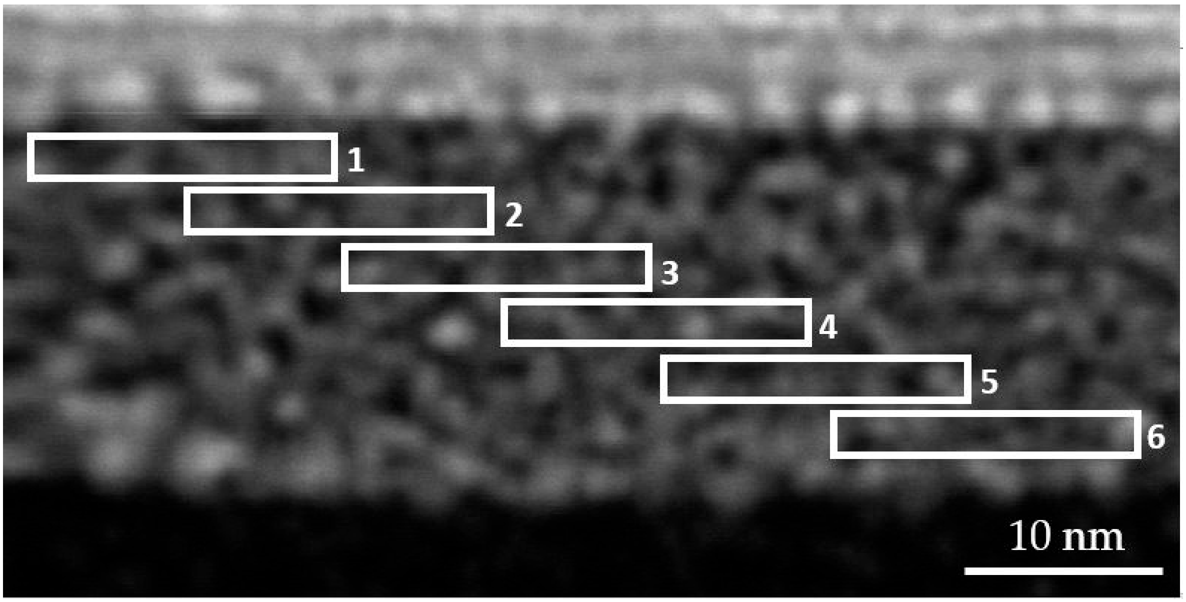

Figure S9. STEM images of the Pt-C cryo-deposit grown under optimized conditions at 30 keV Ga^{+} irradiation. The white rectangles correspond to the areas where EDS experiments were carried out to study the composition along the thickness.

Table S4. Data corresponding to atomic percentages of C, Pt and Ga of the Pt-C cryo-deposit grown under optimized conditions at 30 keV Ga^{+} irradiation.

**Area**	**C (atomic %)**	**Pt (atomic %)**	**Ga (atomic %)**
1	86.71 ± 2.06	13.28 ± 2.76	0 ± 0
2	88.12 ± 2.05	11.87 ± 2.57	0 ± 0
3	88.27 ± 1.69	11.72 ± 1.88	0 ± 0
4	88.54 ± 1.50	11.37 ± 1.86	0.16 ± 0.07
5	88.26 ± 1.45	11.73 ± 1.83	0 ± 0
6	86.97 ± 2.34	13.03 ± 3.30	0 ± 0

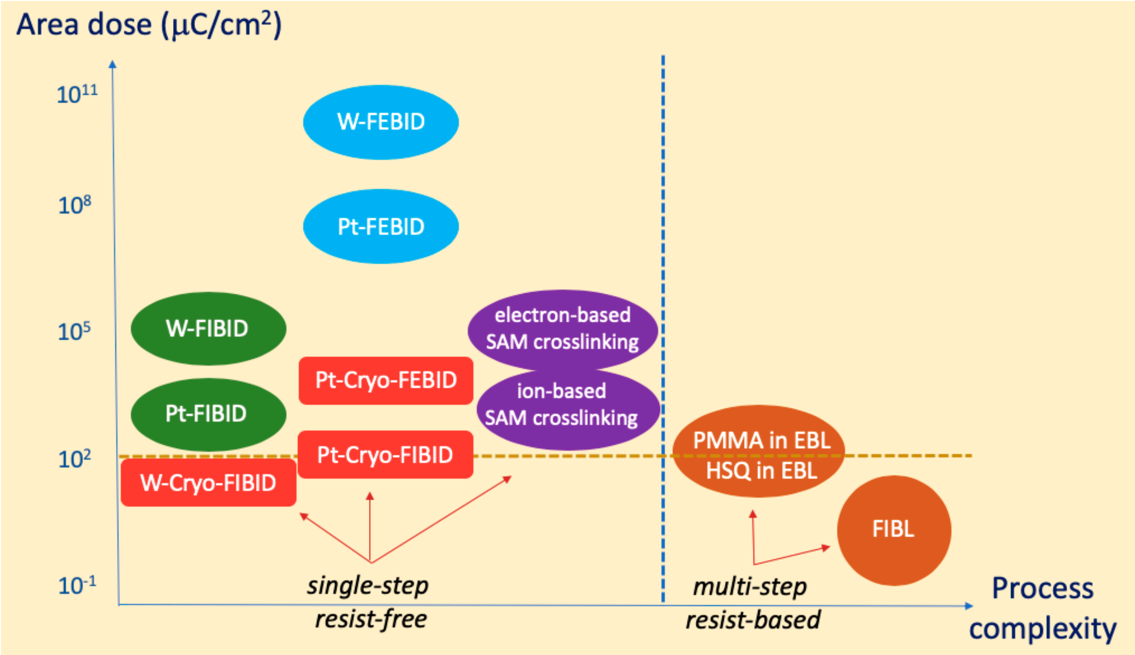

Figure S10. Comparison of charge-particle-based lithography techniques in terms of the required irradiation dose. The cryo-FIBID deposits are those that require the lower irradiation doses per area among the single-step techniques. SAM stands for self-assembled monolayer, PMMA and HSQ are two popular resists, and FIBL stands for focused ion beam lithography. Adapted and modified from De Teresa, J.M. et al., Micromachines 2019 [2].
